# BRAZILIAN AND ARGENTINEAN MULTICENTRIC STUDY IN THE SURGICAL MINIMALLY INVASIVE TREATMENT OF PILONIDAL CYST

**DOI:** 10.1590/0102-672020190001e1447

**Published:** 2019-10-21

**Authors:** Carlos Ramon Silveira MENDES, Luciano Santana de Miranda FERREIRA, Leonardo SALIM

**Affiliations:** 1 Department of Coloproctology, Santa Izabel Hospital, Salvador, BA, Brazil;; 2 Parque Clinic, Coloproctologia Rosario, Rosario, Argentina

**Keywords:** Laparoscopy, Pilonidal cyst, Minimally invasive surgical procedures, Laparoscopia, Cisto pilonidal, Procedimentos cirúrgicos minimamente invasivos

## Abstract

**Background:**

The pilonidal cyst is an infection of the skin and the subcutaneous tissue, secondary to a chronic inflammation with a greater frequency in the sacrococcygeal region, and associated to the presence of hair. The treatment is eminently surgical.

**Aim:**

To demonstrate the endoscopic treatment of pilonidal cyst.

**Method:**

Prospective study with 67 patients who had as surgical indication the diagnosis of pilonidal cyst. They were submitted to a surgical procedure from June 2014 to March 2018. The equipment used was the Meinero fistuloscope, a shutter, a monopolar electrode, a brush and endoscopic forceps.

**Results:**

Of the 67 patients, 67% (n=45) were male and 33% (n=22) female, with a mean age of 25 years (17-45). Surgical time in average was 40 min (20-120) and mean healing time of four weeks (3-12). Surgical complications were presented in 7% cases (n=5) and recurrences in 9% (n=6).

**Conclusion:**

The endoscopic treatment of the pilonidal cyst is feasible and presents good surgical results.

## RESUMO

**Racional:**

O cisto pilonidal é infecção da pele e do tecido subcutâneo, secundário à inflamação crônica, com maior frequência na região sacrococcígea, e associado à presença de pelos nesta região. O tratamento é eminentemente cirúrgico.

**Objetivo:**

Demonstrar os resultados do tratamento endoscópico de cisto pilonidal.

**Método:**

Estudo prospectivo, com 67 pacientes que tiveram como indicação cirúrgica o diagnóstico de cisto pilonidal. Os equipamentos utilizados foram o fistuloscópio Meinero, um obturador, um eletrodo monopolar, uma escova e pinça endoscópica.

**Resultados:**

Dos 67 pacientes 67% (n=45) eram homens e 33% (n=22) mulheres, com média de idade de 25 anos (17-45). O tempo cirúrgico teve com média 40 min (20-120) e o tempo médio de cicatrização de quatro semanas (3-12). Complicações cirúrgicas ocorreram em 7% da amostra (n=5) e recidivas da doença em 9% (n=6).

**Conclusão:**

O tratamento endoscópico do cisto pilonidal é viável e apresenta bons resultados cirúrgicos.

## INTRODUCTION

Pilonidal cyst is a well-known skin and subcutaneous tissue disease, occurring predominantly in men with a mean age of 30 years and an incidence of 26 cases per 100,000^18^. The disease presents complex symptoms, characterized by asymptomatic conditions and painful lesions located in the sacrococcygeal region^6^. The cause is not fully elucidated; however, some risk factors such as obesity, inadequate personal hygiene, family history and long sitting periods are associated with a higher occurrence of the disease^19^.

The treatment is essentially surgical, with a wide variety of techniques including removal of the cyst by means of flap procedures, as seen in the modified Karydakis and Limberg^14^ methods. In addition, other less invasive techniques such as curettage of the cavity with application of phenol^4^ or even the use of laser are commonly used^2^ ^,^ ^8^ ^,^ ^16^. Less invasive techniques are an alternative to methods of surgical excision, presenting advantages such as less postoperative pain, early return of the patient to his activities and reduced scarring^13^.

The minimally invasive endoscopic treatment of pilonidal cyst (EPSiT) proposed by Meinero et al^11^ is based on the treatment of anal fistula by means of video, using a fistuloscope, a shutter, a monopolar electrode, brush and forceps. The technique is subdivided into two stages characterized by the diagnosis phase and the operative phase. Diagnosis is designed to identify and characterize the cyst as well as to identify secondary cavities containing abscesses^11^ ^,^ ^18^. Alternatively Milone (2014)^15^ modified the technique using hysteroscope and saline.

The objective of this study was to demonstrate the effectiveness of minimally invasive endoscopic treatment.

## METHODS

This study was approved by the research ethics committees in the Argentinian and Brazilian institutions, and the patients provided the informed consent form before the surgical procedure.

### Characteristics of the study

This is a prospective study, carried out from June 2014 to March 2018, comprising patients with symptomatic pilonidal cyst who were admitted to Rosario Provincial Hospital in Santa Fe, Argentina, and Santa Izabel Hospital in Salvador, Brazil.

### Surgical technique

**FIGURE 1:**
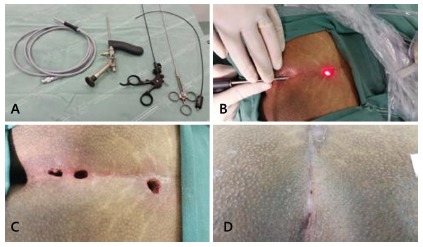
A) Karl-Storz fistuloscope; B) exploration of the cyst; C) final surgical aspect of the procedure; D) postoperative after 15 days

The endoscopic technique was performed according to the method idealized by Meinero et al. (2014)^11^, using the Meinero fistuloscope (Karl Storz GmbH - Tuttlingen, Germany, Figure 1A). The patients underwent spinal anesthesia and the procedure was started in the pronated position, with the buttocks separated with the aid of adhesives, and the surgeon positioned between the patient’s legs.

The procedure begins with the identification and characterization of cyst extension, as well as secondary cavities by insertion of the fistuloscope with infusion of glycine or mannitol into the spontaneous opening of the cyst (Figure 1B). The cyst is then opened with the aid of jet of these liquids in order to identify hairs and eliminate damaged tissues. The hairs present in the tract are removed with the aid of the forceps and the granular tissue is treated by means of the monopolar electrode^13^. All granular tissue is destroyed and removed, the hairs removed, and then the tract is cleaned and left open cavity to facilitate drainage (Figures 1C and 1D).

## RESULTS

The characteristics of the patients and the results obtained are shown in Table 1.

**TABLE 1:** Patient characteristics and results obtained

Variable	n (%)
Age years)	31±14
Gender	
Male	45 (67)
Female	22(33)
Average operation time	40 min
Average healing time (weeks)	4±4
Complications	5 (7)
Recurrences	6(9)

Between June 2014 and March 2018, 67 patients were selected, of which 67% were men and with a mean age of 31 years (17-45). The surgical procedure lasted on average 40 min; the patients were discharged on the same day after the procedure and the mean healing time was four weeks (3-12).

Major complications were not observed; however, five cases had minor complications such as prolonged bleeding and pain. The cure rate was 91%, and five patients had recurrence, and no other case of treatment failure or cyst persistence.

## DISCUSSION

Although pilonidal cyst was described more than 150 years ago^5^, and although the treatment is mostly surgical, there are several surgical techniques described in the literature that include excision of the cyst, flap techniques and more recently minimally invasive techniques^9^. Stauffer and colleagues (2018)^17^ through a systematic review identified at least 14 therapeutic strategies for the treatment of cyst and its recurrence.

In this scenario, Meinero et al^11^ employed the technique of video-assisted anal fistula treatment for the treatment of pilonidal disease, in order to avoid one of the major drawbacks of the procedures that aim to remove the infected area by excision, which is the healing of the surgical wound, open or closed. In both cases, the postoperative period necessitates dressing, which increases the time required for healing, in addition to causing pain^10^ ^,^ ^11^.

The endoscopic technique for the treatment of pilonidal cyst causes less postoperative pain, faster healing and less time to return to daily activities. In addition, it is highly efficient in cases of cyst recurrence.

Endoscopic treatment requires shorter operative time compared to excision methods; Limberg’s flap technique takes an average of 54 min, while that of Karydakis^1^ 48 min (Table 2). The use of the laser demonstrated even shorter surgical time^16^; however, there is greater relapse and treatment failure compared to other techniques.

**TABLE 2:** Therapeutic approaches for the treatment of pilonidal cyst

Author	Sample/gender male	Technique	Operative time (min)	Healing process (days)	Recurrence
Milone et al (2014)^15^	27/19	EPSiT	45±18	NI	1
Bali et al (2015)^1^	71	Limberg	54	22,12±8.69	0
		Karydakis	48	24,08±6.59	0
Pappas & Christodoulou (2018)^16^	237/183	SiLaT	24 (20-30)	47 (30-70)	7 (2.9%)

SiLaT=sinus laser therapy; EPSiT= minimally invasive treatment of pilonidal cyst; NI=not informed

The endoscopic technique has advantages over the less invasive ones that are performed blindly, which explains the greater occurrence of relapses. Endoscopic treatment is favored by the observation of the interior of the cyst, allowing the surgeon to identify the location of the hairs, as well as damaged tissues, contributing to the higher success rate^9^.

The time required for healing after the EPSiT technique was lower than other surgical approaches, presenting even fewer complications. According to Bernier et al. (2015)^3^ about 10-30% of patients undergoing more than one surgical treatment may progress to chronicity. Endoscopic treatment is associated with a low occurrence of recurrence^15^, especially when compared to less invasive techniques^7^.

The complications associated with EPSiT treatment observed in this study were 7%, with self-limiting bleeding in three patients and two reports of pain for a prolonged period, requiring the use of analgesics. No patient demonstrated complications such as necrosis or seroma during follow-up. Prolonged pain requiring analgesics was described in two (22%) patients by Chia et al (2015)^5^ and by Meinero et al^11^ in 9.7%. In this way the complications observed in this study are comparable or better to the other experiences in the literature^16^.

According to Umesh et al (2018)^20^ excision techniques may compromise the sacral fascia and be associated with increased morbidity and increased healing time; on the other hand, the use of the fistuloscope reduces these circumstances, and it presents smaller scars.

Among the several treatments for the pilonidal cyst, the EPSiT technique presents itself as a safe alternative; however, its use depends on specific equipment, which may limit it^13^. Milone et al. (2014)^14^ described a similar procedure using a hysteroscope for performing the video-assisted operation. This procedure also uses saline solution to aid the distension of the cyst.

Dodaro and Renda (2014)^7^ clarify that the Meinero fistuloscope can also be used to treat anal fistulas, in addition to pilonidal cyst, which contributes to reduce the costs and learning curve required to perform the technique.

This technique, pioneered in Brazil and Argentina, represents a safe and reproducible alternative for the treatment of the pilonidal cyst, also allowing the patient to resume his daily activities in a short period of time and in a more aesthetic way, as it results in few scars when compared to other treatments.

## CONCLUSION

Endoscopic treatment for pilonidal cyst treatment demonstrated great safety and efficiency. The technique offers benefits such as good results, reduced recovery time, and low rate of complications.
